# ﻿Two new species of *Hymenagaricus* (Agaricales, Agaricaceae) from Oman, based on morphology and molecular phylogeny

**DOI:** 10.3897/mycokeys.105.113591

**Published:** 2024-04-17

**Authors:** Shah Hussain, Moza Al-Kharousi, Dua’a Al-Maqbali, Arwa A. Al-Owaisi, Rethinasamy Velazhahan, Mohamed N. Al-Yahya’ei, Abdullah M. Al-Sadi

**Affiliations:** 1 Oman Animal and Plant Genetic Resources Center (Mawarid), Ministry of Higher Education, Research and Innovation, P.O. Box 515, P.C. 123, Muscat, Oman Sultan Qaboos University AlKhoud Oman; 2 Department of Plant Sciences, College of Agricultural and Marine Sciences, Sultan Qaboos University, PO Box 34, AlKhoud 123, Oman Oman Animal and Plant Genetic Resources Center Muscat Oman

**Keywords:** Dhofar, diversity, taxonomy, termite mounds, two new taxa

## Abstract

*Hymenagaricus* has small to medium-sized mushrooms and the cap surface with squamulose pellicles, consisting of hymeniform or pseudoparenchymatous cells and yellowish-brown basidiospores. The species of *Hymenagaricus* are very similar to those of *Xanthagaricus* and it is extremely difficult to differentiate the species of both genera in the field. However, phylogenetically, both the genera are clearly distinct. In this study, we describe two new species of *Hymenagaricus*, i.e. *H.wadijarzeezicus* and *H.parvulus* from the southern part of Oman. Species descriptions are based on a combination of morphological characteristics of basidiomata and phylogenetic analyses of three gene regions: internal transcribed spacer (ITS1-5.8S-ITS2 = ITS), the large subunit of nuclear ribosomal DNA (28S) and translation elongation factor one alpha (EF-1α). Full descriptions, micrographs and illustration of anatomical features, basidiomata photos and phylogenetic analyses results of the new taxa are provided. Morphological comparisons of new taxa with similar species and a key to species included in the phylogenetic analyses are also provided.

## ﻿Introduction

Three species previously in AgaricussubgenusConioagaricus, *Agaricushymenopileus*, *A.alphitochrous* and *A.nigrovinosus*, were placed in a new and separate genus called *Hymenagaricus* Heinem. by Heinemann in 1981. This taxonomic change was likely due to the distinct features observed in the cap of these species during different stages of their development. At the young stage, the pilei are entirely covered with a pellicle, but as they mature, the pellicle is disrupted, leaving a single large squamulose pellicle at the centre of the pileus. The squamules are composed of hymeniform or pseudoparenchymatous cells, which set these species apart from others within the genus *Agaricus* (Heinemann 1981). The genus *Hymenagaricus* was typified by *H.hymenopileus* (Heinem.), belonging to the family Agaricaceae Chevall. (Heinemann 1981).

Species of *Hymenagaricus* are saprotrophic in nature and are mostly distributed in the Palaeotropical Regions. Members of this genus are recognised by the squamulose pellicle on the pileus surface that mostly consists of hymeniform cells or pseudoparenchymatous tissues, yellow to yellowish-brown basidiospores and the absence of both pleurocystidia and clamp connections (Heinemann and Little Flower 1984; Reid and Eicker 1995; Little Flower et al. 1997; Hosen et al. 2017; Al-Kharousi et al. 2022a). The number of known species in the genus is 17 (Hussain et al. 2018; Kumla et al. 2021, 2023; Syed et al. 2023).

Phylogenetically, species of *Hymenagaricus* are intermixed with the monotypic genus *Heinemannomyces* Watling (Hosen et al. 2017; Hussain et al. 2018). This intermixing may be due to limited molecular data available for the previously-described species of *Hymenagaricus*. However, morphologically, both genera can be differentiated. *Heinemannomyces* with single species *H.splendidissimus* Watling, distributed in southeast Asia, has medium-sized basidiomata, with woolly fibrillose cap surface, composed of pseudoparenchymatous cells and the spore print is leaden-grey to dark blue (Watling 1998).

Four species of Agaricaceae, namely *Agaricusarabiensis* S. Hussain & Al-Sadi, *Micropsalliotaventricocystidiata* Al-Sadi & S. Hussain, *Xanthagaricusappendiculatus* Al-Sadi & S. Hussain and *X.omanicus* Al‐Kharousi, Al‐Sadi & S. Hussain have recently been described from Dhofar Region, Oman (Al-Kharousi et al. 2022a, 2022b; Hussain et al. 2022). However, no *Hymenagaricus* species has been reported from the country.

During the years 2022–23, macrofungal exploration missions were conducted in the Dhofar Region, in which we collected ten (10) collections of *Hymenagaricus*. Morphological characterisation and multigene (ITS, 28S, EF-1α) phylogenetic analyses revealed that the 10 collections represent two new species, which are described in this study.

## ﻿Materials and methods

### ﻿Study sites and field sampling

The specimens were collected in the Dhofar Region, located in the south of the Sultanate of Oman. The region experiences a monsoon-influenced climate with a distinct wet season known as the Khareef, which occurs from June to early September (Bookhagen et al. 2005). During this time period, the moist and cool air from the Indian Ocean is drawn in by the southwest monsoon, bringing significant rainfall into the region, which is extremely rare in the rest of the Arabian Peninsula, including Oman. This seasonal variation supports a diverse ecosystem and separates Dhofar from the arid desert conditions that prevail in the Arabian Peninsula (El-Sheikh 2013). The Khareef season triggers the growth of various plants and trees, including frankincense trees, creating a lush and vibrant landscape where a number of saprotrophic mushrooms can flourish (Al-Kharousi et al. 2022a).

In the current study, mushroom specimens were collected from three localities (Wadi Naheez, Wadi Jahaneen, Wadi Jarzeez) of the Dhofar Region, in the months of August–September 2022 to 2023. The specimens were photographed in the field and field characteristics such as the shape, colour, size and smell of basidiomata were noted. The samples were dried using a fruit dehydrator with temperature adjusted at 45 °C (Hu et al. 2022). After drying, the specimens were kept in zip lock plastic bags and stored at -80 °C for two weeks to kill all the insects/eggs/larvae. After the cold temperature treatment, the samples were characterised morpho-anatomically and phylogenetically. All the samples are deposited in Oman Animal and Plant Genetic Resources Center (Mawarid), AlKhoud, Muscat, Sultanate of Oman.

### ﻿Morphological investigation

For microscopic study, handmade sections were made from lamellae, cap and stipe surfaces and annulus. Thin small sections were initially mounted in 5% aqueous potassium hydroxide (KOH) (w/v) and then re-hydrated in 1% aqueous Cong red (w/v) for a more obvious appearance. Microscopic features such as the size, shape and colour of basidiospores, basidia, cheilocystidia, pellicle structure, veil and annulus morphology were studied under a compound microscope (ECLIPSE Ni-U, Nikon Co., Ltd., Japan). For size measurements of these structures, Piximetre (http://ach.log.free.fr/Piximetre/) was used. For the morphological terminology, Vellinga and Noordeloos (2001) was followed.

### ﻿DNA extraction, PCR amplification and sequencing

Genomic DNA was extracted from dried specimens using X-AMP DNA reagent kit (Dubuque, Iowa, USA), following the manufacturer’s protocol. A volume of 200 µl X-AMP DNA reagent was taken in an Eppendorf tube containing the sample (approximately 5–15 mg of gills) and incubated for 15 minutes at 70 °C. After cooling, 2 µl solution from the sample was used as a DNA template directly for polymerase chain reaction (PCR) without any further treatment. We amplified three gene regions, including the internal transcribed spacer (ITS), the large subunit of nuc rDNA (28S) and the translation elongation factor 1 alpha (EF-1α) gene. The primer combinations were: ITS1F and ITS4 for ITS (White et al. 1990; Gardes and Bruns 1993), LR0R and LR5 for 28S (Vilgalys and Hester 1990; White et al. 1990), EF1-983 and EF1-1567R for EF-1α (Rehner and Buckley 2005). PuReTaqTM Ready-To-Go PCR beads (GE Healthcare UK Limited, Buckinghamshire, UK) were used for PCR amplification. We added 1.0 µl of each primer (10 µmol/l), 2 µl DNA template and 22 µl Nuclease free water to each bead. For ITS amplification, the PCR conditions were optimised as: initial denaturation at 95 °C for 5 min, followed by 35 cycles of denaturation at 95 °C for 30 s, annealing at 54 °C for 45 s and extension at 72 °C for 1 min. For the 28S and EF-1α regions, only the annealing temperature was optimised, 52 °C for 28S and 60 °C EF-1α, respectively (Hussain et al. 2022). The PCR products were purified and then sequenced from Macrogen Inc. © (Seoul, Republic of Korea) bidirectionally using the same primers.

### ﻿Sequence alignment and phylogenetic analyses

Consensus sequences were created from the forward and reverse primer reads of the newly-generated ITS, 28S and EF-1α sequences using BioEdit v.7.0.9.0 (Hall 1999). We performed BLAST searches for the newly-generated sequences; only ITS and 28S regions showed maximum similarity with *Hymenagaricus* species. In the case of EF-1α sequences, the BLAST search revealed *Heinemannomyces* sp. (ZRL185) is the most similar species because, in GenBank, no EF-1α sequences of *Hymenagaricus* are available. This is the reason that we used only ITS and 28S sequences in the phylogenetic analyses. A combined ITS-28S dataset was constructed from the sequences used in the recent studies of *Hymenagaricus* (Mwanga and Tibuhwa 2014; Hosen et al. 2017; Kumla et al. 2021, 2023; Syed et al. 2023). The final ITS-28S dataset was comprised of 27 specimens, including 26 ITS and 20 28S sequences (Table 1). *Agaricuscampestris* L. (LAPAG370) was used as the outgroup taxon. Sequences were aligned using MAFFT v.7 (Katoh et al. 2019) and visually inspected using BioEdit v.7.0.9.0 (Hall 1999). Maximum Likelihood (ML) and Bayesian Inference (BI) methods were used for the phylogenetic analyses. Maximum Likelihood (ML) phylogeny was performed with RAxML-HPC BlackBox, implemented on CIPRES Science Gateway (Miller et al. 2010; Stamatakis 2014). The best model (GTR+F+I+G4) was chosen following jModelTest2 (Darriba et al. 2012). Branch support for the ML phylogeny was executed with 1000 bootstrap replicates. For BI analyses, we used BEAST v.1.8.2 (Drummond et al. 2012). The combined ITS-28S alignment was converted to XML datafile using BEAUti v.1.8.2 (Bayesian Evolutionary Analysis Utility; Drummond et al. (2012)). A Birth-Death Incomplete Sampling speciation model (Stadler 2009) was selected. Four independent runs were performed with BEAST on XSEDE tool on the CIPRES Science Gateway (Miller et al. 2010). Resulting log files were checked in Tracer (Rambaut et al. 2014) for effective sample size (ESS) values. All ESS values were well over 200. Tree files were combined in LogCombiner v.1.8.2 (Drummond and Rambaut 2007). A maximum clade credibility (MCC) tree was obtained using the TreeAnnotator v.1.8.2 (Drummond and Rambaut 2007). The ML bootstrap (BT) percentage ≥ 70 and BI posterior probabilities (PPs) ≥ 0.80, respectively, were considered significant. For phylogenetic tree visualisation, FigTree v.1.4.2 (Rambaut 2012) was used and the tree was annotated using Adobe Illustrator CC2019. The alignment file is submitted to TreeBase (http://purl.org/phylo/treebase/phylows/study/TB2:S30802).

**Table 1.** Taxa included in the molecular phylogenetic analyses.

Species	Origin	Voucher number	GenBank accession		Reference
			ITS	28S	
*Agaricuscampestris*	Spain	LAPAG370	KM657927	KP739803	Parra et al. (2016)
*Heinemannomycessplendidissimus*	China	Q. Zhao 2591	KY039571	KY039576	Yang and Ge (2017)
*Hei.splendidissimus*	China	Z.W. Ge2540	KY039570	KY039575	Yang and Ge (2017)
*Hei.splendidissimus*	China	GDGM46633	MF621038	MF621039	Hosen et al. (2017)
*Hei.splendidissimus*	China	GDGM46634	–	MF621040	Hosen et al. (2017)
*Hei.splendidissimus*	Thailand	ecv3586	HM488760	HM488769	Vellinga et al. (2011)
*Hei.* sp.	Thailand	ZRL185	KT951346	KT951527	Zhao et al. (2016)
*Hymenagaricusardosiaecolo*r	Togo	LAPAF9	JF727840	–	Zhao et al. (2011)
*H.ardosiaecolor*	Tanzania	Z4	KM360160	–	Mwanga and Tibuhwa (2014)
H.cf.kivuensis	Burundi	BR6089	KM982454	–	Mwanga and Tibuhwa (2014)
***H.wadijarzeezicus***	**Oman**	**JRZ2-22-015**	**OR613000**	**OR613018**	**This study**
***H.wadijarzeezicus***	**Oman**	**JRZ2-22-013**	**OR612999**	**OR613019**	**This study**
***H.wadijarzeezicus***	**Oman**	**NHZ-22-019**	**OR612997**	**OR613020**	**This study**
***H.wadijarzeezicus***	**Oman**	**JHN-22-019**	**OR612998**	**OR613021**	**This study**
***H.wadijarzeezicus***	**Oman**	**JRZ-22-005**	**OR612996**	**OR613022**	**This study**
*H.pakistanicus*	Pakistan	FAK196	OP082405	–	Syed et al. (2023)
*H.pakistanicus*	Pakistan	FAK195	OP082404	–	Syed et al. (2023)
***H.parvulus***	**Oman**	**JRZ-22-004**	**OR612994**	**OR613017**	**This study**
***H.parvulus***	**Oman**	**JRZ2-22-002**	**OR612995**	–	**This study**
*H.siamensis*	Thailand	SDBR-CMUWP038	OP837533	OP836600	Kumla et al. (2023)
*H.siamensis*	Thailand	SDBR-CMUNK1508	OP836301	OP836385	Kumla et al. (2023)
*H.saisamornae*	Thailand	ZRL3103	KM982450	KM982452	Kumla et al. (2021)
*H.saisamornae*	Thailand	SDBRCMUNKNW0474	MW349605	MW349603	Kumla et al. (2021)
*H.saisamornae*	Thailand	SDBR-CMUNK0369	MW345912	MW345917	Kumla et al. (2021)
*H.saisamornae*	Thailand	SDBR-CMUNK0567	MW349602	MW349604	Kumla et al. (2021)
*H.saisamornae*	Thailand	LD2012186	KM982451	KM982453	Kumla et al. (2021)
*H.* sp.	Pakistan	LAH35329	OQ998344	–	Usman (2018)
*H.* sp.	Thailand	CA833	JF727858	–	Zhao et al. (2011)

## ﻿Results

### ﻿Phylogenetic analyses

**Figure 1.**
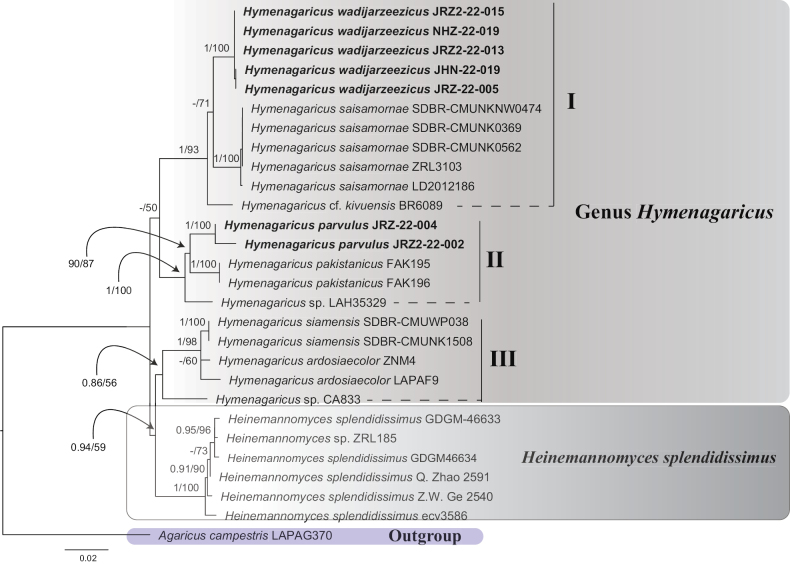
Maximum Likelihood phylogeny of *Hymenagaricus* and *Heinemannomyces*, based on combined ITS-28S sequence data, with *Agaricuscampestris* as the outgroup taxon. Values above the node represent ML bootstrap percentages and BI posterior probabilities; the new species are represented in bold fonts.

In this study, 19 new sequences (7 ITS, 6 28S and 6 EF-1α) were generated from our collections of *Hymenagaricus*. There were no EF-1α sequences of the genus available in GenBank; therefore, only combined ITS-28S sequences were used in the final data matrix. The final ITS-28S dataset was comprised of 1516 characters including 1272 constant sites, 141 informative sites and 103 uninformative sites. The topology of trees revealed similar patterns in both ML and BI methods; therefore, the phylogeny inferred from ML analysis is presented here with values from both BT and PPs in Fig. 1. In both ML and BI analyses, six specimens of *Heinemannomyces* formed a basal group, sharing a clade with *Hymenagaricus* species. This clade is weakly supported in ML analyses and well supported with BI (BT 59%, PPs 0.94). However, the subclade representing *Heinemannomyces* specimens is strongly supported in both analyses (BT 100%, PPs 1). Species of *Hymenagaricus* are distributed in three clades. Clade-I with good statistical support (BT 93%, PPs 1) consisted of three species, the new species *Hymenagaricuswadijarzeezicus*, *H.saisamornae* J. Kumla & N. Suwannarach and H.cf.kivuensis Heinem. Each of these three species has its unique position, confirming their unique identity. Similarly, clade-II was strongly supported (BT 100%, PPs 1), with three taxa, *H.pakistanicus* M.F. Syed & M. Saba, the new species *H.parvulus* and an unnamed species *H.* sp. (LAH35329). The third is a subclade in the clade consisting of *Hymenagaricus* and *Heinemannomyces* taxa. This subclade consisted of *H.siamensis* J. Kumla, W. Phonrob, N. Suwannar & S., Lumyong, *H.ardosiaecolor* Heinem. and an unnamed species *H.* sp. (CA833). However, the two specimens (LAPAF9, Z4) representing *H.ardosiaecolor*, were recovered with different branch lengths. This variation in branch length could be the result of using only ITS sequences of *H.ardosiaecolor* in the phylogenetic analyses.

### ﻿Taxonomy

#### ﻿ Hymenagaricus wadijarzeezicus

Taxon classificationFungiAgaricalesAgaricaceae

Al‐Sadi, Al-Yahya’ei, A. Al-Owaisi & S. Hussain sp. nov.

13253B07-3074-51BD-93B8-51361F369CDE

850249

Figs 2 , 3 , 4

##### Diagnosis.

The new species *Hymenagaricuswadijarzeezicus* can be differentiated from other species of the genus by its unique whitish woolly veil, covering both the cap and the stipe surfaces.

##### Holotype.

Sultanate of Oman: Dhofar, Salalah, Wadi Jarzeez, on termite mounds, under the trees of *Anogeissusdhofarica*, 11 August 2022, S. Hussain, A. Al-Owaisi & Al-Yahya’ei, JRZ2-22-013 (holotype Mawarid-JRZ2-22-013), GenBank accession: ITS = OR612999, 28S = OR613019, EF-1α = OR729599.

##### Etymology.

The specific epithet ‘*wadijarzeezicus*’ refers to the valley Jarzeez in the south of Oman, where the holotype was found.

##### Description.

***Basidiomata*** small to medium-sized. ***Pileus*** 30–80 mm in diam., at the young stage, broadly ovoid to parabolic, covered completely by a smooth, pale brownish pellicle; at mature stage, pulvinate to convex, pellicle disrupting except at the centre where it is retained as one large, smooth, brownish squamule, surface is woolly, covered with whitish, strigose to villose or floccose veil towards the margin; margin appendiculate with long, whitish, fibrils of veil. ***Context*** dark pinkish on cutting, 3–5 mm thick at the pileus centre. ***Lamellae*** free, pale pinkish at young stage, at mature stage greyish-pink to brownish, ventricose, up to 3 mm wide, densely crowded, with 1–3 series of lamellulae. ***Stipe*** 30–60 × 5–10 mm, equal, with a slightly bulbous base, with root-like rhizoid structure at the base, annulus floccose, concolorous to veil; stem covered with floccose veil below the annulus, smooth above the annulus, context pinkish on cutting, fistulose. ***Smell*** pleasant. ***Taste*** not recovered.

***Basidiospores*** (6.5)7.0–8.0(8.5) × (4.0)4.5–5.5(6.0) µm, average size 7.5 × 5.0 µm, Q = 1.4–1.6, av. Q = 1.5; ellipsoid to broadly ellipsoid, yellowish to dark brown, smooth, thick-walled, apiculus visible, germ-pore not observed. ***Basidia*** 20–25 × 7–9 µm, on average 22.5 × 8.0 µm, clavate to cylindrical, smooth, hyaline in KOH, mostly tetrasporic, rarely bisporic. ***Cheilocystidia*** 16–23 × 7–9 µm, on average 19.5 × 8.0 µm, ellipsoid to subclavate, smooth, thin-walled, hyaline in KOH. ***Pleurocystidia*** absent. ***Lamellar trama*** regular, with 4–6.6 µm diam., cylindrical to inflated, thin-walled, hyaline hyphae. ***Subhymenium*** consisted of subglobse to irregular cells, measuring 12–18 µm diam. ***Pellicle*** is a hymeniform, consisting of chains of two or three elements, measuring 13–17 × 10–16 µm each element, globose to subglobose or ovoid, hyaline, or pale yellowish, smooth, thin-walled, these chains of elements attached to inflated hyphae with encrusted walls. ***Pileus veil*** is a cutis to ixocutis, consisting of elongated or cylindrical elements, easily detached, hyaline, thin-walled, each element measuring 13–45 × 6–9 µm. ***Annulus*** is an intricate trichoderm, composed of hyaline hyphae, 6–8 µm diam., cylindrical, constituted by short elements, constricted at septa, easily disarticulated. ***Stipe veil*** similar to pileus veil. ***Clamp connections*** absent in all tissues.

**Figure 2.**
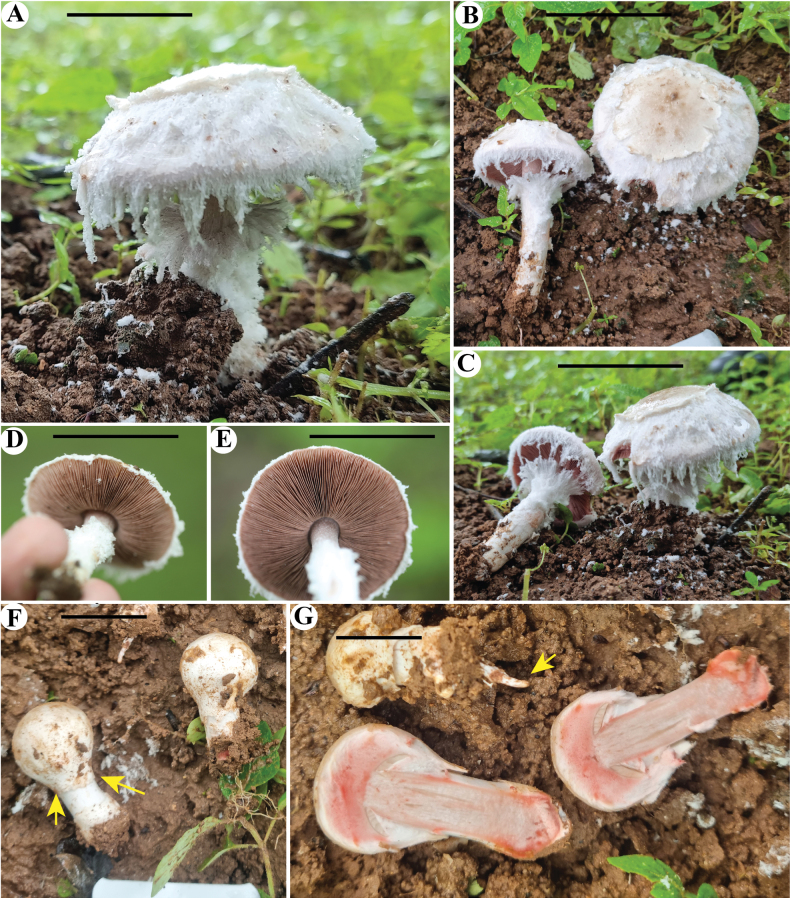
Basidiomata of *Hymenagaricuswadijarzeezicus***A–C** holotype collection (JRZ2-22-013) **D, E** NHZ-22-019 **F** young fruiting bodies where the cap is entirely covered by pellicle represented by arrows (JRZ2-22-015) **G** context changed into pinkish on cutting, the arrow represents the root-like rhizoid (JRZ2-22-015). Scale bars: 20 mm.

##### Habit, habitat, and distribution.

Occurring in July to early September, as saprotrophic, solitary or scattered in small groups, on or near the termite mounds, under the trees of *Anogeissusdhofarica*. Currently only known from southern Oman.

**Figure 3.**
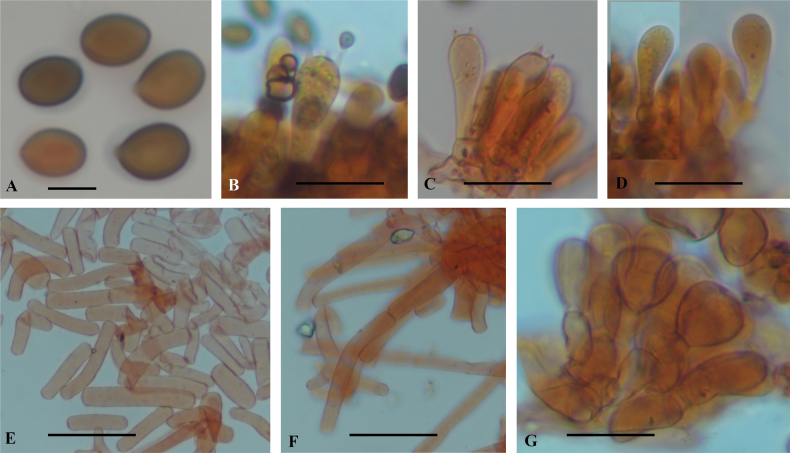
Light microscopy of anatomical features of *Hymenagaricuswadijarzeezicus* (based on holotype collection JRZ2-22-013) **A** basidiospores **B, C** basidia **D** cheilocystidia **E** annulus **F** veil elements **G** pellicle structure. Scale bars: 10 µm (**A**); 15 µm (**B–D**); 15 µm (**E–G**).

##### Additional specimens examined.

Sultanate of Oman: Dhofar, Salalah, Wadi Naheez, on termite mounds, under the trees of *Anogeissusdhofarica*, 07 August 2022, S. Hussain, A. Al-Owaisi, Al-Yahya’ei & Al-Sadi, NHZ-22-019 (Mawarid-NHZ-22-019), GenBank accession: ITS = OR612997, 28S = OR613020, EF-1α = OR729602; Wadi Jarzeez, under the trees of *Anogeissusdhofarica*, 08 August 2022, S. Hussain, A. Al-Owaisi, Al-Yahya’ei & Al-Sadi, JRZ-22-005 (Mawarid-JRZ-22-005), GenBank accession: ITS = OR612996, 28S = OR613022, EF-1α = OR729603; Wadi Jaheen, under the trees of *Anogeissusdhofarica*, 10 August 2022, S. Hussain, A. Al-Owaisi, Al-Yahya’ei & Al-Sadi, JHN-22-019 (Mawarid-JHN-22-019), GenBank accession: ITS = OR612998, 28S = OR613021, EF-1α = OR729600; Wadi Jarzeez, on termite mounds, under the trees of *Anogeissusdhofarica*, 11 August 2022, S. Hussain, A. Al-Owaisi & Al-Yahya’ei, JRZ2-22-015 (Mawarid-JRZ2-22-015), GenBank accession: ITS = OR613000, 28S = OR613018, EF-1α = OR729601; Wadi Gogob, on termite mounds, under the trees of *Anogeissusdhofarica*, 22 August 2023, S. Hussain & Al-Yahya’ei, GOB-23-008 (Mawarid-GOB-23-008); Sahalanawt, on termite mounds, 27 August 2023, S. Hussain & Muhammad Salim, Sahalanawt-23-001 (Mawarid-Sahalanawt-23-001); Tetam, on termite mounds, 30 August 2023, S. Hussain & Amer Qattan, Tetam-23-001 (Mawarid-Tetam-23-001).

**Figure 4.**
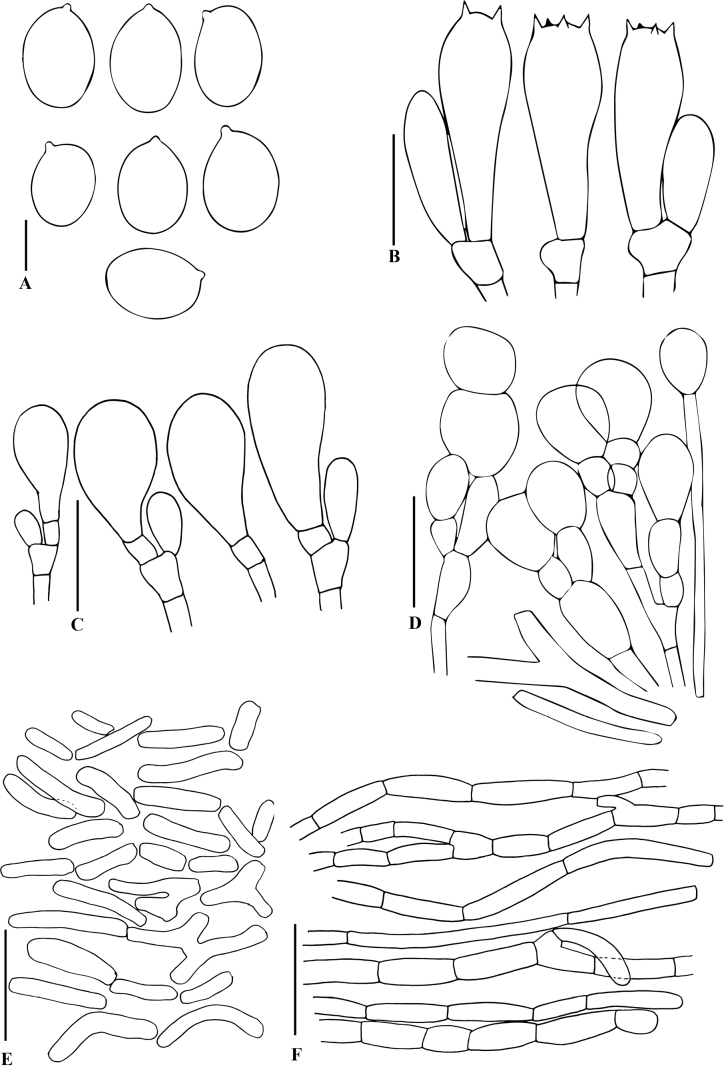
Line drawings of anatomical features of *Hymenagaricuswadijarzeezicus* (based on holotype collection JRZ2-22-013) **A** basidiospores **B** basidia **C** cheilocystidia **D** pellicle structure **E** annulus **F** veil elements. Scale bars: 10 µm (**A**); 15 µm (**B–D**); 15 µm (**E, F**).

##### Notes.

The new species *Hymenagaricuswadijarzeezicus* with medium-sized basidiomata, can be distinguished from the known species of the genus by its remarkable woolly cap and stipe surfaces. In *Hymenagaricus*, there are four species with a cap diameter of 50 mm or above, which are: Hymenagaricuscf.kivuensis, *H.mlimaniensis* Mwanga & Tibuhwa, *H.ardosiaecolor* and *H.alphitochrous* (Berk & Broome) Heinem. *Hymenagaricuswadijarzeezicus* is the 5^th^ species with a cap diameter above 50 mm. None of these species has a woolly basidiomata surface, except *Hymenagaricuswadijarzeezicus*.

In ML phylogeny, the most similar species to the new species *H.wadijarzeezicus* is *H.saisamornae*. *Hymenagaricussaisamornae* is a recently described species from Thailand, with substantially smaller basidiomata (10–25 mm cap diam.), pileus surface covered with minute brownish squamules, stipe smooth to finely whitish squamulose and smaller basidiospores (5.5–7.0 × 4.0–4.5 µm; Kumla et al. (2021)). Similarly, Hymenagaricuscf.kivuensis and *H.mlimaniensis*, both African species, shared medium-sized pileus with *H.wadijarzeezicus*. Hymenagaricuscf.kivuensis has pileus of 50–100 diam., with smaller basidiospores (4.0–6.5 × 3.0–4.5 µm), narrower basidia (16–20 × 4.5–6) and broader hymeniform cells (Pegler 1977; Heinemann 1984). *Hymenagaricusmlimaniensis* has a broadly umbonate, reddish-brown disc, with sparsely squamulose surface and smaller basidiospores (4.0–7.0 × 3.5–4.5 µm; Mwanga and Tibuhwa (2014)) than *H.wadijarzeezicus* (7.0–8.0 × 4.5–5.5 µm). *Hymenagaricussiamensis* differs from *H.wadijarzeezicus* by its smaller basidiomata with brownish cap, measuring 22–32 mm diam., squamules consisting of pseudoparenchymatous cells (Kumla et al. 2023). Another small-sized species *Hymenagaricuspakistanicus* with pileus 24–30 mm diam., covered with dark brownish squamules at the cap centre, smaller basidiospores (5.0–6.0 × 3.5–5.0 µm) and a pseudoparenchymatous pellicle (Syed et al. 2023).

#### ﻿ Hymenagaricus parvulus

Taxon classificationFungiAgaricalesAgaricaceae

Al‐Kharousi, Al‐Sadi, Al-Yahya’ei, & S. Hussain sp. nov.

82BEAF0B-C6A8-5E01-9615-B20090130FC6

850248

Figs 5 , 6 , 7

##### Diagnosis.

The new species *Hymenagaricusparvulus* can be differentiated from other species of the genus by its small-sized, creamy basidiomata, umbonate pileus covered with appressed pellicle.

##### Holotype.

Sultanate of Oman: Dhofar, Salalah, Wadi Jarzeez, on termite mounds, under the trees of *Anogeissusdhofarica*, 8 August 2022, S. Hussain, A. Al-Owaisi, Al-Yahya’ei & Al-Sadi, JRZ-22-004 (holotype Mawarid-JRZ-22-004), GenBank accession: ITS = OR612994, 28S = OR613017, EF-1α = OR735176.

##### Etymology.

The specific epithet ‘*parvulus*’ refers to the small-sized basidiomata of the new species.

##### Description.

***Basidiomata*** small-sized. ***Pileus*** 15–25 mm in diam., at young stage globose to parabolic, surface floccose squamulose, squamules light pinkish to creamy, with appressed pellicle at the centre, margin appendiculate; at mature stage cap convex to hemispherical with the broadly umbonate disc, with appressed, pale brownish pellicle at the disc, surface finely floccose squamulose, squamules pale creamy to light greyish, margins striate, just exceeding the lamellae; context membranous, pinkish on cutting. ***Lamellae*** free, pale pinkish to brownish, ventricose, sparsely crowded, with 1–2 series of lamellulae. ***Stipe*** 25–35 × 2–5 mm, equal, annulus cortinate, concolorous to squamules; stem surface creamy, covered with finely floccose squamules below the annulus, smooth above the annulus, context pinkish on cutting, fistulose. ***Smell*** pleasant. ***Taste*** not recorded.

**Figure 5.**
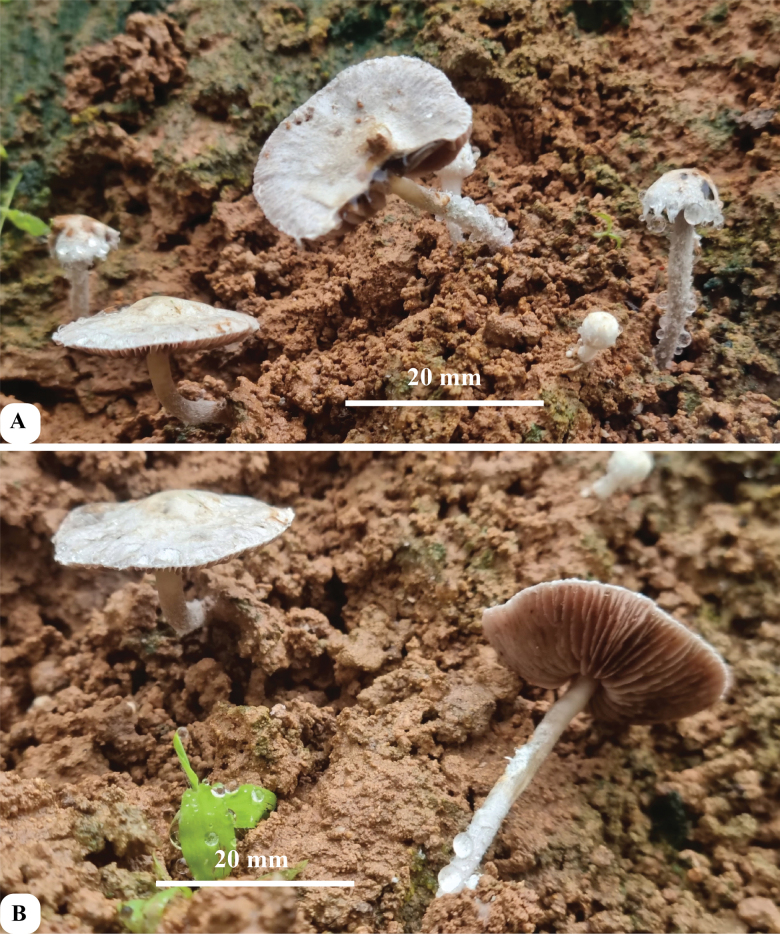
Basidiomata of *Hymenagaricusparvulus* (based on holotype collection JRZ-22-004) **A** mature and young basidiomata **B** mature basidimata.

***Basidiospores*** 5.0–6.5 × 4.0–4.5 µm, average size 6.0 × 4.2 µm, Q = 1.3–1.5, av. Q = 1.4; ellipsoid to broadly ellipsoid, yellowish to dark brown, smooth, thick-walled, apiculus visible, germ-pore not observed. ***Basidia*** 16.5–22.5 × 6.5–8.5 µm, on average 19.0 × 7.5 µm, clavate to cylindrical, smooth, hyaline in KOH, tetrasporic. ***Cheilocystidia*** 19–25 × 9–11 µm, on average 22 × 10 µm, clavate to broadly clavate, often turning to one side, with multiseptate base, smooth, thin-walled, hyaline in KOH. ***Pleurocystidia*** absent. ***Subhymenium*** consisting of cylindrical to elongated cells, measuring 6–9 µm diam. ***Pellicle*** is a hymeniform, consisting of chains of several elements, each element measuring 14–22 × 12–17 µm, globose to subglobose or ovoid, hyaline or pale yellowish, smooth, thin-walled; these chains of elements attached to inflated hyphae with encrusted walls. ***Veil*** is a cutis to ixocutis, consisting of elongated or cylindrical elements, not easily detached, hyaline, thin-walled, with terminal element fusiform with papillate end, each element measuring 15–18 × 5–7 µm. ***Annulus*** is an intricate trichoderm, composed of hyaline hyphae, 4–7 µm diam., cylindrical, constituted by short elements, constricted at septa and easily disarticulated. ***Clamp connections*** absent in all tissues.

**Figure 6.**
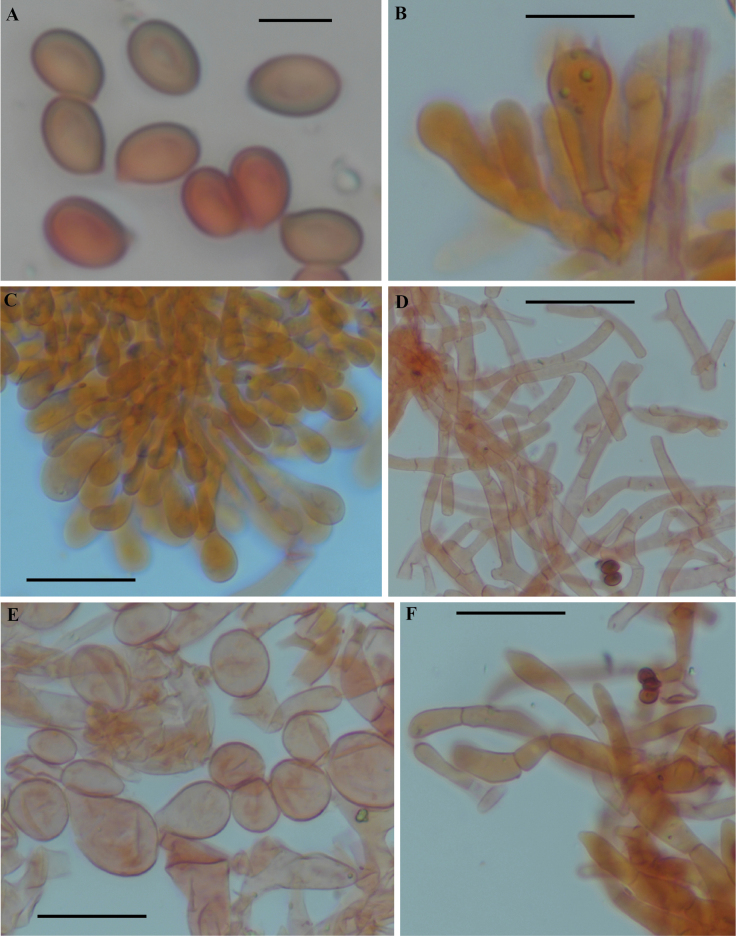
Light microscopy of anatomical features of *Hymenagaricusparvulus* (based on holotype collection JRZ-22-004) **A** basidiospores **B** basidia **C** cheilocystidia **D** annulus **E** pellicle structure **F** veil elements. Scale bars: 5 µm (**A**); 10 µm (**B, C**); 15 µm (**D–F**).

##### Habit, habitat and distribution.

Fruiting body formation occurs in early August to early September, saprotrophic, scattered in small groups, found on termite mounds. Currently only known from southern Oman.

**Figure 7.**
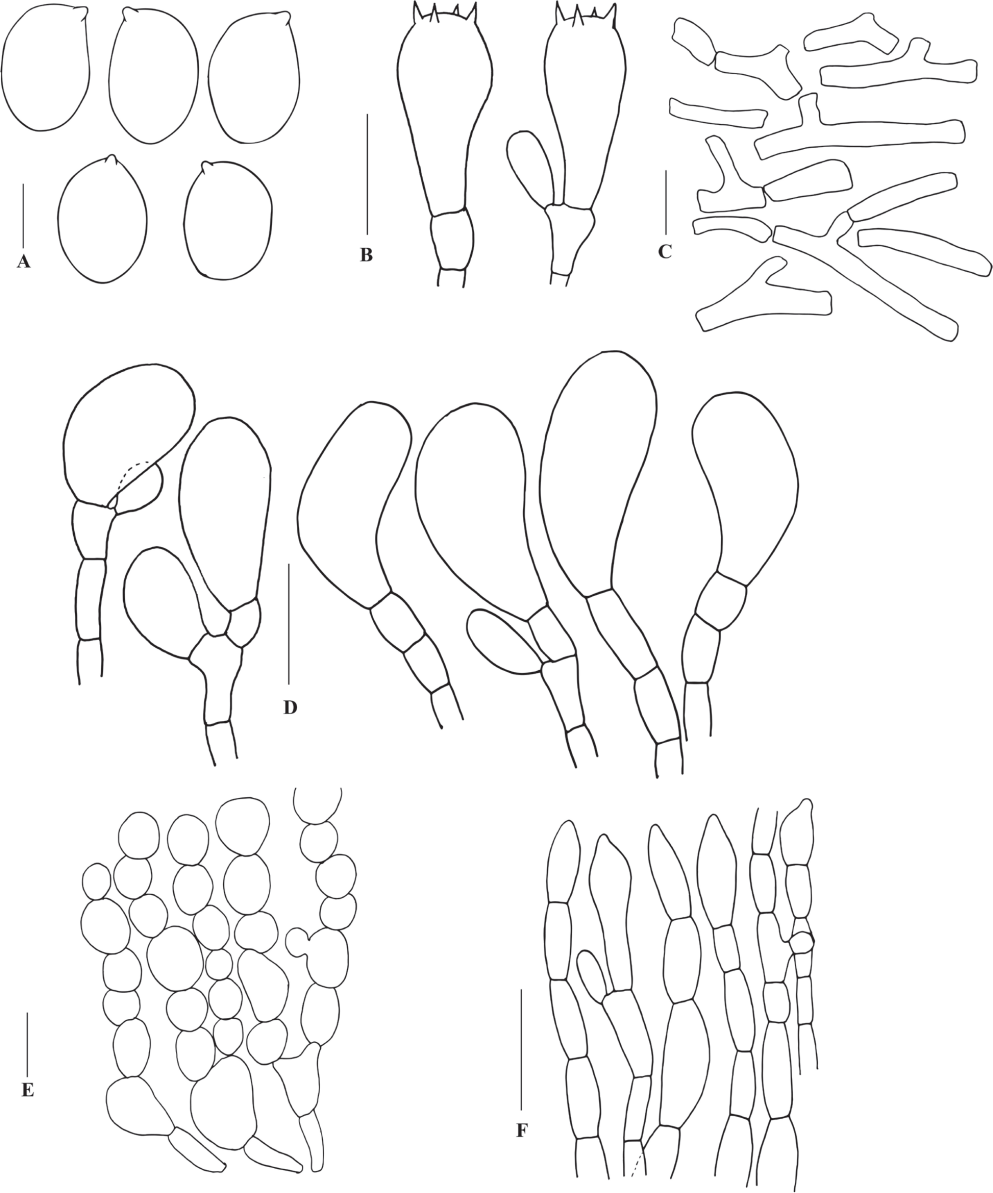
Line drawings of anatomical features of *Hymenagaricusparvulus* (based on holotype collection JRZ-22-004) **A** basidiospores **B** basidia **C** annulus elements **D** cheilocystidia **E** pellicle structure **F** veil elements. Scale bars: 5 µm (**A**); 10 µm (**B, D**); 15 µm (**C, E, F**).

##### Additional specimens examined.

Sultanate of Oman: Dhofar, Salalah, Wadi Jarzeez, on termite mounds, under the trees of *Anogeissusdhofarica*, 11 August 2022, S. Hussain, A. Al-Owaisi, Al-Yahya’ei & Al-Sadi, JRZ2-22-002 (Mawarid-NHZ-22-002), GenBank accession: ITS = OR612995.

##### Notes.

*Hymenagaricusparvulus* is a small, cream-coloured species, differentiated from other species of the genus by its whitish to pale pinkish floccose squamules on pileus and stipe surfaces with a broadly umbonate centre. *Hymenagaricusparvulus* shares basidiomata size and basidiospores morphology with *H.pakistanicus*. However, *H.pakistanicus* can be differentiated from the new species by its caesptiose fruiting habit, pileus with pinkish to brownish squamulose pellicle, consisting of pseudoparenchymatous cells (Syed et al. 2023). *Hymenagaricussaisamornae* differs from the new species by its smaller pileus (up to 15 mm diam. Vs. 15–25 mm of *H.parvulus*), covered with brownish pellicles and larger basidiospores (5.5–7.0 × 4–4.5 µm; Kumla et al. (2021)). *Hymenagaricussiamensis*, another small-sized species is distinguished from the new species by its pinkish-brown cap, pellicle comprised of pseudoparenchymatous cells and larger basidiospores (6.5–8.0 × 4.0–5.0 µm; Kumla et al. (2023)). Similarly, *Hymenagaricuscanoruber* (Berk. & Br.) Heinem. & Little Flower, known from India and Sri Lanka, is characterised by a small-sized pileus (15–25 mm diam.), with greyish-brown squamules, hymeniform pellicle and smaller basidiospores (4.6–5.7 3.5–4.3 µm; Heinemann and Little Flower (1984)). *Hymenagaricuspallidodiscus* D.A. Reid & Eicker, the smallest mushroom in the genus with pileus diam. up to 11 mm, covered with brownish squamules and smaller basidiospores (4.2–5.4 × 3.1–3.8 µm; Reid and Eicker (1999)). *Hymenagaricuscylindrocystis* Heinem. & Little Flower another small-sized species, has been reported in Singapore and India, with a brownish cap, larger basidiospores (6.4–8.4 × 4.5–5.6 µm) and a pseudoparenchymatous pellicle (Heinemann 1956; Heinemann and Little Flower 1984). Hymenagaricuscf.kivuensis and *H.wadijarzeezicus* with their medium-sized pilei can be easily differentiated from *H.parvulus*.

## ﻿Discussion

Species of *Hymenagaricus* and *Xanthagaricus* are morphologically very similar and it is extremely difficult to differentiate the species of these genera in the field. However, in most species of *Xanthagaricus*, the cap surface is covered with small, brownish to purplish scales. These scales are concentrated at the pileus centre, while a large central, undisrupted scale at the cap centre has been observed in the most species of *Hymenagaricus*. Phylogenetically, both the genera are clearly distinct.

Phylogenetically, the species of *Hymenagaricus* are closely related to the monotypic genus *Heinemannomyces*. Morphologically, both these genera are clearly distinct. Species of *Hymenagaricus* have a squamulose cap surface and these squamules consist of hymeniform or pseudoparenchymatous cells and yellowish-brown basidiospores. The monotypic genus with single species *Heinemannomycessplendidissimus* has a brownish to greyish-red pileus, covered with a finely woolly veil and greyish to dark bluish basidiospores (Watling 1998; Hosen et al. 2017).

In our phylogenetic analyses, the specimens representing *Heinemannomyces* formed a basal group. Species of *Hymenagaricus* were recovered in three groups. One group consisted of *Hymenagaricuswadijarzeezicus*, the new species, *H.saisamornae* and H.cf.kivuensis. In this group, *Hymenagaricussaisamornae* with small-sized basidiomata intermix with H.cf.kivuensis and *H.wadijarzeezicus* both with medium-sized basidiomata. Similarly, another group consisting of *Hymenagaricuspakistanicus*, *H.parvulus* the new species and unnamed species *H.* sp. (LAH35329). All these taxa, including the unnamed species, have a small fruiting body. The third group consists of *Hymenagaricusardosiaecolor* (medium-sized basidiomata) and *H.siamensis*, the small-sized species.

Both basidiomata size and pellicle structure are species delimitation characters in the genus *Hymenagaricus*. Based on our analyses, we can predict that these characters could be used in the future for infrageneric classification of the genus.

The two new species, *Hymenagaricuswadijarzeezicus* and *H.parvulus*, were collected in the Dhofar Region, located in the southern part of Oman. *Hymenagaricuswadijarzeezicus* is medium-sized and *H.parvulus* is a small-sized species. Both are widespread in the Region, under the trees of *Anogeissusdhofarica*. It is interesting to note that both collections of *H.parvulus* (JRZ-22-002, JRZ2-22-004) and several collections of *H.wadijarzeezicus* (NHZ-22-019, JRZ2-22-013, JRZ2-22-015, GOB-23-008, Sahalanawt-23-001, Tetam-23-001) were found on termite mounds. However, we did not find any study reporting the association of *Hymenagaricus* with termites. However, secotioid fungal genus *Podaxis* Desv. in the family Agaricaceae has an apparent relationship with termites (Conlon et al. 2016). It will be interesting to study the relationships of these mushrooms with termites.

Several species of Agaricaceae were recently reported from the Dhofar Region (Al-Kharousi et al. 2022a, 2022b; Hussain et al. 2022). It is evident that the area is rich in the diversity of Agaricaceae, including the genus *Hymenagaricus*. More new species of dark-spored agarics are likely occurring in the area.

### ﻿Taxonomic key to the species of *Hymenagaricus*

A taxonomic key to the species of *Hymenagaricus* included in our phylogenetic analyses is presented below. This key is based on cap diameter (small-sized with cap less than 40 mm in diam. and medium-sized with cap ranging from 50–100 mm in diam.) and pellicle structure either hymeniform or pseudoparenchymatous cells.

**Table:** 

1	Basidiomata small-sized, pileus diam. below 40 mm	**2**
–	Basidiomata medium-sized, pileus diam. up to 100 mm	**5**
2	Fruiting bodies solitary or gregarious	**3**
–	Fruiting bodies appear in cluster (casepitose), pileus 20-30 mm diam., pellicle consisted of pseudoparenchymatous cells	***Hymenagaricuspakistanicus***
3	Pileus whitish or pinkish-brown, pellicle consisted of hymeniform cells	**4**
–	Pileus brownish, pellicle consisted of pseudoparenchymatous cells	***H.siamensis***
4	Pileus 15–25 mm diam., whitish to creamy, pellicle smooth, finely appressed	***H.parvulus***
–	Pileus 10–15 mm diam., pinkish to brownish, squamulose pellicle	***H.saisamornae***
5	Pileus covered with brownish squamules	**6**
–	Pileus and stipe surfaces covered with whitish woolly fibrils	***H.wadijarzeezicus***
6	Pileus diam. 40–60 mm, basidiospores 5.8–6.5 × 3.9–4.5 µm	***H.ardosiaecolor***
–	Pileus diam. up to 100 mm, hymeniform squamules	**H.cf.kivuensis**

## Supplementary Material

XML Treatment for Hymenagaricus wadijarzeezicus

XML Treatment for Hymenagaricus parvulus

## ﻿Additional information

### Author ORCIDs

Shah Hussain https://orcid.org/0000-0002-5772-7206

Rethinasamy Velazhahan https://orcid.org/0000-0002-9263-4371

Mohamed N. Al-Yahya’ei https://orcid.org/0000-0002-9516-5339

Abdullah M. Al-Sadi https://orcid.org/0000-0002-3419-8268

### Data availability

All of the data that support the findings of this study are available in the main text.
